# RADtyping: An Integrated Package for Accurate *De Novo* Codominant and Dominant RAD Genotyping in Mapping Populations

**DOI:** 10.1371/journal.pone.0079960

**Published:** 2013-11-21

**Authors:** Xiaoteng Fu, Jinzhuang Dou, Junxia Mao, Hailin Su, Wenqian Jiao, Lingling Zhang, Xiaoli Hu, Xiaoting Huang, Shi Wang, Zhenmin Bao

**Affiliations:** Key Laboratory of Marine Genetics and Breeding, College of Marine Life Sciences, Ocean University of China, Qingdao, Shandong Province, China; Temasek Life Sciences Laboratory, Singapore

## Abstract

Genetic linkage maps are indispensable tools in genetic, genomic and breeding studies. As one of genotyping-by-sequencing methods, RAD-Seq (restriction-site associated DNA sequencing) has gained particular popularity for construction of high-density linkage maps. Current RAD analytical tools are being predominantly used for typing codominant markers. However, no genotyping algorithm has been developed for dominant markers (resulting from recognition site disruption). Given their abundance in eukaryotic genomes, utilization of dominant markers would greatly diminish the extensive sequencing effort required for large-scale marker development. In this study, we established, for the first time, a novel statistical framework for *de novo* dominant genotyping in mapping populations. An integrated package called RADtyping was developed by incorporating both *de novo* codominant and dominant genotyping algorithms. We demonstrated the superb performance of RADtyping in achieving remarkably high genotyping accuracy based on simulated and real mapping datasets. The RADtyping package is freely available at http://www2.ouc.edu.cn/mollusk/ detailen.asp?id=727.

## Introduction

Genetic linkage maps are indispensable tools in genetic, genomic and breeding studies. A high-resolution linkage map is exceptionally valuable in many applications such as fine-scale quantitative trait locus (QTL) mapping, characterization of recombination hotspots, comparative genome analysis, genome scaffolding and marker-assisted selection. The advent of next-generation sequencing technologies has greatly stimulated the development of a variety of genotyping by sequencing methods that enable simultaneously discovering and genotyping of thousands of single nucleotide polymorphisms (SNPs). In particular, RAD (restriction-site associated DNA) has gained popularity for linkage map construction [1], and several methods with simpler library preparation protocols have been developed, such as 2b-RAD [2] and ddRAD [3]. With increasing demands for application of the RAD method in poorly-studied organisms, several tools such as Stacks [4], RApiD [5], RADtools [6] and iML [7] have been developed to analyze RAD data *de novo* (i.e., in the absence of a reference genome). However, these tools are being predominantly used for scoring codominant markers; while for dominant markers, which are scored as “presence” or “absence” due to the disruption of recognition sites, available tools basically only output the raw count of tag presence or absence. For these tools, the accuracy of dominant genotype calls remains unclear. No experimental validation has been performed to determine what percentage of the observed tag presence/absence polymorphism is really due to restriction site heterozygosity but not the variation of sequencing depth. A statistical framework for *de novo* dominant genotyping remains to be established. It has been shown that dominant markers can provide a large amount of additional genotypic information (e.g., accounting for ∼40% of total markers in the threespine stickleback; [8]), the utilization of which would greatly diminish the extensive sequencing effort required for large-scale marker development. The implications of dominant marker variation have been explored in several recent studies [9]–[11]. In the present study, we established, for the first time, a novel statistical framework for *de novo* dominant genotyping in linkage mapping studies. An integrated package called RADtyping was developed, which could achieve accurate *de novo* codominant and dominant genotyping in mapping populations. The performance of RADtyping was thoroughly evaluated using both simulated and real mapping datasets.

## Results and Discussion

### Overview of the RADtyping methodology

**Figure 1:**
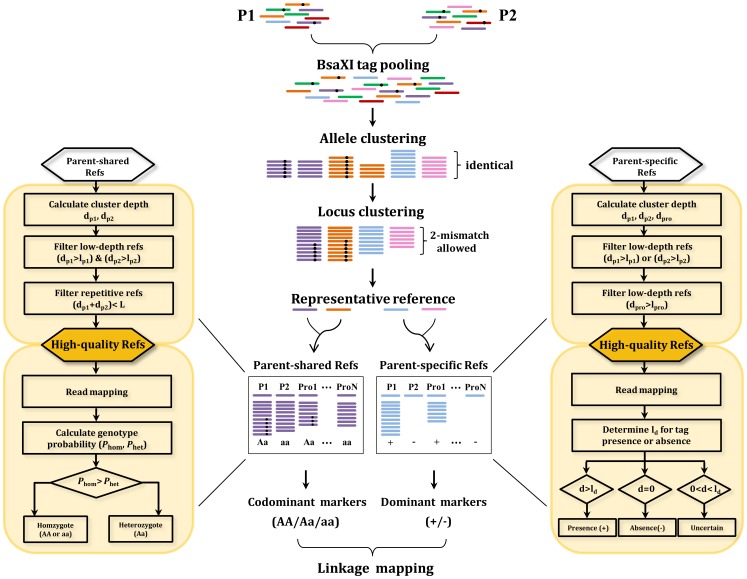
An overview of the RADtyping approach for *de novo* codominant and dominant genotyping in a mapping population. Representative reference sites are obtained by assembling parental sequencing reads into “locus” clusters. These sites are further classified into parent-shared and parent-specific sites for subsequent codominant and dominant genotyping. Main principles of codominant and dominant genotyping algorithms are shown in flowcharts, and more details are described in the Methods section.

The principle of RADtyping is outlined below (also shown in Figure 1) and a full description of the genotyping algorithms is available in the Methods section.

#### Representative reference reconstruction

Reference sites are reconstructed using sequencing data from both mapping parents. Briefly, all pre-processed reads from two mapping parents are combined and assembled into exactly matching read clusters (i.e. representing individual alleles), and then these “allele” clusters are further merged into “locus” clusters by allowing certain mismatches. A collection of consensus sequences from all the “locus” clusters comprises the representative reference sites. These sites are further classified into parent-shared and parent-specific sites for subsequent codominant and dominant genotyping, respectively.

#### Codominant genotyping

To obtain high-quality reference sites, parent-shared reference sites are first filtered by excluding sites that are either not supported by parental reads in sufficient depth or derived from repetitive genomic regions. Here the iML algorithm recently developed by our group is adopted to exclude repetitive sites from genotyping, the performance of which has been thoroughly evaluated [7]. Once the high-quality reference sites are determined, sequencing reads from the two parents and their progeny are separately mapped to those sites. For each locus, posterior probabilities are calculated for two possible genotypes (i.e., homozygote or heterozygote) and then a likelihood ratio test is performed to determine the most likely genotype.

#### Dominant genotyping

Unlike codominant markers, dominant markers are scored as “presence” or “absence” to reflect whether a recognition site is intact or disrupted. Similar to codominant genotyping, parent-specific reference sites that are not supported by parental reads in sufficient depth are first filtered out. In addition, reference sites that are not sequenced to sufficient depth in the progeny are also excluded to avoid incorrect “absence” calls from these low-coverage sites. Sequencing reads are then mapped to the high-quality reference sites obtained, and the “absence” or “presence” of each site is determined using the threshold *l_d_* to prevent incorrect “presence” calls from sites with misaligned reads.

### RADtyping performance on simulation data

**Figure 2:**
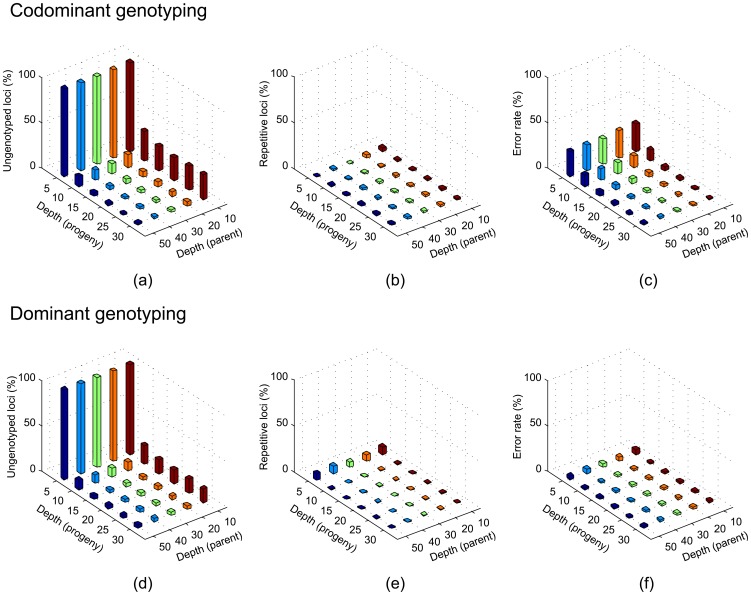
Evaluation of the performance of RADtyping using a pseudo F_1_ mapping population. The simulated population was created by a crossing of two *Arabidopsis* plants with predefined SNPs in their genomes and progeny were subject to *in silico* sequencing together with their parents at different sequencing depths with sequencing errors enabled. *De novo* codominant and dominant genotyping was evaluated in three key aspects: genotype coverage (a, b), removal of repetitive sites (b, e), and genotyping accuracy (c, f).

The performance of RADtyping was first evaluated using *in silico* sequencing datasets generated from a pseudo *Arabidopsis* F_1_ mapping population (see methods for details). The aims of our simulation analysis were (i) to evaluate the performance of RADtyping in three key aspects (i.e., genotype coverage, removal of repetitive sites and genotyping accuracy), and (ii) to help devise a cost-effective sequencing strategy for linkage mapping studies by balancing sequencing cost and genotyping accuracy. The simulation results showed that with increased sequencing depth for parents and their progeny, the percentage of ungenotyped loci rapidly decreased and reached a “stable” level at the sequencing depth combination of ≥20× for parents and ≥15× for progeny where a majority of target loci (>93% for codominant loci and >96% for dominant loci) could be readily genotyped (Figure 2a,d, Table S1a,d). The high-quality reference sites reconstructed for genotyping almost exclusively derived from unique genomic regions (e.g. >98% at the sequencing depth of ≥10× for both parents and progeny; Figure 2b,e, Table S1b,e), suggesting that repetitive sites could be efficiently filtered out by our genotyping algorithms. The rate of genotyping error gradually decreased with the increase of sequencing depth. For codominant genotyping, genotyping accuracy could reach ∼97% at the sequencing depth of 20× for both parents and progeny (Figure 2c, Table S1c), while for dominant genotyping, ∼98% could be achieved at a much lower sequencing depth (10×; Figure 2f, Table S1f), suggesting that dominant loci could be more reliably genotyped than codominant loci when the average sequencing depth was low. In addition, our simulation results suggest that a minimal sequencing depth of 20× for both parents and progeny should meet the desired level of genotyping accuracy in large-scale linkage mapping studies.

### RADtyping performance on real data

The performance of RADtyping was further evaluated using a sequencing dataset generated from an F_1_ mapping population of Zhikong scallop, *Chlamys farreri* [12]. The sequencing depth for progeny ranged from 13.4 to 23.8 with an average of 16.75, whereas the parents were sequenced to a much deeper depth (70∼80×). Clustering parental reads resulted in 181,625 representative reference sites. After a series of quality-filtering steps, 117,113 parent-shared and 35,799 parent-specific sites composed the list of high-quality reference sites. These reference sites contained 92% of the unique sites inferred from a preliminary reference genome we recently generated for *C. farreri* (870 Mb, equivalent to ∼70% genome coverage; available at http://ipl.ouc.edu.cn/fuxiaoteng/cf_SRA_data; [12]), suggesting that unique sites were well represented in the obtained high-quality reference sites. In total, 7,458 polymorphic markers were identified (Table 1), of which 6,842 that were heterozygous in at least one parent were suitable for linkage analysis, including 2,196 codominant and 4,646 dominant markers. Obtaining more dominant markers than codominant markers should be related to the low-sequencing coverage of progeny. RAD sites with low read depth are more likely to be genotyped by dominant algorithm than codominant algorithm. For codominant RAD sites, when we count all sites that have read coverage in at least 80% of progeny (regardless of their genotyping status), the number of codominant markers increase to 8,679, representing 1.7 times the number of dominant markers (5,251). Genotyping accuracy was further evaluated by amplicon (Sanger) sequencing of eight codominant and eight dominant markers for two parents and four progeny. The average validation rate was 96% and 97% for the codominant and dominant markers, respectively (Table 2). Particularly, all 2b-RAD genotypes in parents could be validated by the Sanger method, suggesting that genotyping accuracy can be substantially improved through deep sequencing (∼50×). For the validated dominant markers, SNPs that disrupted the recognition sites were also confirmed (Table 3).

**Table 1:** Summary of polymorphic markers obtained by 2b-RAD sequencing of a *C. farreri* mapping population.

	Segregation pattern	Total marker no.^a^	Marker no. in accord with Mendelian segregation^b^	Mapped marker no.
Codominant marker	(AA×aa) or (aa×AA)	203	n.a.	n.a.
	(Aa×aa) or (aa×Aa)	1882	1432	1166
	(Aa×Aa)	314	233	187
Dominant marker	(AA×--) or (--×AA)	413	n.a.	n.a.
	(A-×--) or (--×A-)	n.a.	3216	2453
	(A-×A-)^c^	n.a.	1430	n.a.

a Total marker no. refers to all polymorphic markers reported by RADtyping regardless of whether they follow Mendelian segregation in progeny.

b For dominant markers, only those in accord with Mendelian segregation were scored to ensure the correct assignment of markers to different segregation patterns.

c This segregation type was scored separately apart from the main pipeline.

**Table 2:** Sanger validation of 2b-RAD genotypes.

Maker type	Genotype class	2b-RAD genotype	Validated by Sanger sequencing	Validation rate
**Codominant**				
Parent (depth: 49–77×)	Heterozygote	8	8	100%
	Homozygote	8	8	100%
Progeny (depth: 14–21.2×)	Heterozygote	12	10	84%
	Homozygote	20	20	100%
Total		48	46	96%
**Dominant**				
Parent (depth: 37–63×)	Presence	8	8	100%
	Absence	8	8	100%
Progeny (depth: 13.7–22×)	Presence	13	12	92%
	Absence	8	8	100%
Total		37	36	97%

**Table 3:** Codominant and dominant SNPs confirmed by Sanger-based amplicon sequencing.

Marker	BsaXI tags^a^	Forward primer (5′→3′)	Reverse primer (5′→3′)
**Codominant**			
m119628	TGGTAGGAA**AC**TTTTT**CTCC**TCGT(C/T)CC	GCAGAGTTGGCAAAGGGG	ACACGGCCAGAACCCAGC
f83678	AAACAGTTT**AC**ATGGA**CTCC**CC(T/C)AACA	ACTGCTCCCACCTCTGAC	GTAGTCCCAGTTGCTCCA
m12011	TCAAAGATA**AC**CCTAT**CTCC**(G/A)CTATAG	TTCTGCTTGTCCACACACGACCTCC	ACTGCTGCTGTTTCTTACACTTATG
f47186	CATGG(G/C)GTC**AC**TTGAT**CTCC**CGACAGA	CCCCTTACCTTCACTGT	TGTGACAACACTGACTCG
f79797	TAACCGATG**AC**GAGTA**CTCC**GAAGT(G/A)T	GGTCTGGTACAAACAAATGAC	AGACAGACTGCTTTGCCA
m81459	GCTAACGCC**AC**AAAAA**CTCC**C(C/A)GAGAG	GAAGTTCAAAAGGGAGTA	GAGCAATGTTAGGGCTAA
m386	ACCT(T/G)CGAA**AC**TAATT**CTCC**GAAATGT	ATCAAGCGTCAATATAACCTG	AGAAGCACAACACTGCTGTAC
f12046	TA(T/A)ATAGTT**AC**TGATC**CTCC**AAGATTT	TTAGGTGTAAGTAAGGAC	TTTAGTCGGCTAGTATTG
**Dominant**			
df33179	ACCTGCTTC**AC**AGAAG**CT(C/G)C**TTCGAAT	TCTACCGACCGACGGACTGA	ACTAGTTCCCTGTTCTTTTACTGAT
dm25086	CATTCCACC**AC**CCCAC**C(T/G)CC**CACCCAA	GATAAACGACTGAGTGGAAC	GGTGCGCTAATGGAAATA
df29520	CGTTGCAGA**AC**TCAGG**C(T/A)CC**GCCCCTC	TAACGTAGCGACATCAGG	ATTGAGTTCAGGAGTTTCC
dm27070	CACAAACAC**AC**ATTAA**C(T/C)CC**TGACATT	AACTAAAGCTACCCAGACAC	GACGCTAGATGGATGACA
df4428	CTGATAAGG**AC**CGCTG**CT(C/T)C**CCCTCTC	ATCATTACAGTAACTTCCACTCGGT	ACGGCTGACTACCTGTAAACATTGA
df12778	TTCATTTGA**A(C/T)**TCTCC**CTCC**TTTAATG	ATTACACCTGCATGAACAA	TAATGAACTGTGGGACGC
df9608	TCTACGTAT**A(C/T)**ATTTC**CTCC**CACTCCA	CTGATGGCAAGTTGTATCCAGAATG	CATAATATAAGACCAAATCATCACA
dm25622	CATTGAGCT**(A/T)C**CCAGT**CTCC**AGACCTC	CTTATGCTTACAAAGGAGGT	ATCTAAGTTGTTGGGCAGT

a BsaXI restriction sites are highlighted in bold and SNP alleles are indicated in parentheses.

Currently, it remains difficult to evaluate the accuracy of RAD genotyping tools at a large scale due to lack of a gold standard RAD mapping dataset with pre-known true genotypes (especially for dominant markers). To circumvent this problem, we generated a mapping dataset by 2b-RAD sequencing of replicate libraries that were independently prepared from two scallop parents (*Argopecten irradians irradians* and *Argopecten purpuratus*) and ten of their F_1_ hybrid progeny. Measuring genotyping consistency between these replicate datasets enables providing a good proxy for the overall genotyping accuracy of RADtyping. In total, 5,533 mappable markers were identified by requiring being genotyped in both datasets for at least 80% of progeny, including 1,561 codominant markers and 3,972 dominant markers (present in one parent and absent in another) in accordance with Mendelian segregation. Very high genotyping consistency was revealed between the two replicate datasets with on average 96% for codominant markers (Table 4) and 99% for dominant markers (Table 5), which further substantiates the superb performance of RADtyping in achieving accurate *de novo* codominant and dominant genotyping in mapping populations. The finding of slightly higher consistency for dominant markers than codominant markers coincides with our previous simulation results, i.e, dominant loci can be more reliably genotyped than codominant loci at the same sequencing depth. Note, heterozygous loci showed relatively lower genotyping consistency in progeny than parents (Table 4), which is most likely related to the difference of average sequencing depths between parents (181–235×) and progeny (22–46×).

**Table 4:** Consistency of codominant genotyping on replicate 2b-RAD libraries prepared from two parents and ten progeny.

	Genotyped from Replicate 2			
Genotyped from Replicate 1	Homozygous (Parent)	Heterozygous (Parent)	Homozygous (Progeny)	Heterozygous (Progeny)
Same genotype	1,527	1,578	6,813	5,307
Different, homozygous	0	8	0	401
Different, heterozygous	5	4	150	13
Agreement (%)	99.7%	99.2%	98.1%	92.8%

Note, average sequencing depths for two parents were 181× and 185× in rep1 and 190× and 235× in rep2, while for progeny, they were 37–46× in rep1 and 22–30× in rep2.

**Table 5:** Consistency of dominant genotyping on replicate 2b-RAD libraries prepared from two parents and ten progeny.

	Genotyped from Replicate 2			
Genotyped from Replicate 1	Absent (Parent)	Present (Parent)	Absent (Progeny)	Present (Progeny)
Same genotype	3,972	3,972	14,133	12,915
Different, absent	–	0	–	316
Different, present	0	–	112	–
Agreement (%)	100%	100%	99.2%	97.6%

Note, average sequencing depths for two parents were 181× and 185× in rep1 and 190× and 235× in rep2, while for progeny, they were 37–46× in rep1 and 22–30× in rep2.

### Future directions for RADtyping improvement

In the present study, the performance of RADtyping was evaluated only based on 2b-RAD datasets. Though we expect that RADtyping should be generally applicable to various kinds of RAD data, it remains to be tested. Our *de novo* genotyping algorithms currently assume that RAD data approximately follow a mixed Poisson (or normal) distribution. However, this assumption may not be appropriate for all kinds of RAD data [9]; therefore incorporating alternative distribution models (e.g. negative binomial) seems a better choice to further improve the utility of this program.

Currently, RADtyping only deals with dominant markers showing 1∶1 segregation pattern in progeny, i.e., parental genotypes are A- for one parent and – for another, where - represents an unsequencable allele resulting from a mutation in the restriction site. While for dominant markers showing 1∶2∶1 segregation pattern (i.e., A-×A-), a statistical genotyping approach still needs to be established. The forseeable most challenging step is to accurately distinguish AA from A- especially in cases where deep sequencing is not feasible.

In conclusion, RADtyping enables accurate *de novo* genotyping of codominant and dominant markers in mapping populations, which would greatly facilitate construction of high-resolution linkage maps in organisms lacking extensive genomic resources.

## Materials and Methods

### Simulated and real sequencing data

For simulation analysis, a pseudo F_1_ mapping population composed of 100 progeny was created *in silico* for the model plant species *Arabidopsis thaliana*. Approximately 1% of the BsaXI sites in the *Arabidopsis* genome were randomly chosen as polymorphic sites. For each polymorphic locus, a parental genotype was designated as either homozygote or heterozygote at a rate of 50%, while for progeny, genotypes were randomly generated by conforming to the law of independent recombination. *In silico* sequencing was performed for the pseudo *Arabidopsis* mapping population. Different sequencing depths were evaluated for the parents (10× to 50×) and the progeny (5× to 30×). Each allele was “sequenced” to a depth determined by a draw from a Poisson distribution. For each “sequenced” read, the global error rate, which increased linearly along the sequence, was set to 1%.

Two real sequencing datasets were utilized in this study. The first dataset was retrieved from our recent linkage mapping study for *C. farreri* [12], which was generated by 2b-RAD sequencing of two parents and 96 F_1_ progeny. Briefly, 2b-RAD libraries were prepared by following the protocol developed by Wang et al. [2]. For the parents, standard BsaXI libraries were constructed, while for the progeny, reduced representation (RR) libraries were constructed using adaptors with 5′-NNT-3′ overhangs to target a subset of BsaXI fragments in the genome. 2b-RAD libraries were subject to single end sequencing (1×50 bp) using an Illumina GA-II sequencer. The second dataset was retrieved from our ongoing linkage mapping project for *Argopecten irradians irradians* and *Argopecten purpuratus*, which was generated by 2b-RAD sequencing of an F_1_ hybrid family created by crossing *A. irradians* (♀) and *A. purpuratus* (♂). Similar to the first dataset, standard libraries were constructed for parents and RR libraries were constructed for progeny using adaptors with 5′-NNA-3′ and 5′-NNT-3′overhangs. Replicate libraries were independently constructed for two parents and ten progeny, and then sequenced (1×36 bp) in two separate sequencing runs on an Illumina HiSeq2000 sequencer. All of the 2b-RAD sequences were archived in the SRA database under accession numbers SRA065207 (first dataset) and SRP029614 (second dataset).

### RADtyping methodology

RADtyping is a pipeline program that integrates all custom Perl scripts necessary for implementing *de novo* codominant and dominant genotyping algorithms. RADtyping can deal with both single-end and paired-end RAD sequencing data. The principle of its genotyping strategy is elaborated as follows.

Paternal and maternal reads are first pooled together to assemble into exactly matching read clusters (i.e. representing individual alleles), and then “allele” clusters are further merged into “locus” clusters by allowing two mismatches using the Ustacks program (parameters -m 3, -M 2; [4]). A collection of consensus sequences from all of the “locus” clusters comprises a set of representative reference sites that are further classified into parent-shared and parent-specific sites.

For the parent-shared sites, cluster depth (*d*) approximately follows a mixed Poisson distribution due to the existence of composite clusters:
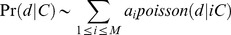
(1)where 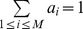 and *M* represents the copy number for repetitive sites. The parameters *C* and *a_1_*-*a_M_* can be estimated from the sequencing data using the expectation-maximization (EM) algorithm. To remove low-quality sites, reference sites are filtered to retain those supported by parental reads in sufficient depth (i.e. the requirements *d_p1_*>*l_p1_* and *d_p2_*>*l_p2_*). The thresholds *l_p1_* and *l_p2_* are determined by:
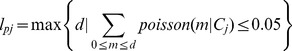
(2)where *C_j_* is the mean sequencing depth of the *j* th parent (j = 1,2). To remove repetitive sites, parent-shared sites are filtered by excluding those with depths larger than *L*. The threshold *L* is determined by:
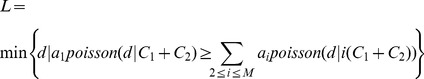
(3)


For the parent-specific sites, low-quality sites (i.e. *d_p1_*<*l_p1_* or *d_p2_*<*l_p2_*) are also removed. In addition, to avoid incorrect “absence” calls from low-coverage sites in the progeny, the reference sites are further filtered to remove those with *d_pro_* less than *l_pro_*, where *d_pro_* is calculated for each site by summarizing all progeny having reads derived from that site, and the threshold *l_pro_* is determined for each site using formula (2).

Once the high-quality reference sites are obtained, sequencing data from the parents and progeny are separately mapped against these sites using SOAP2 software (parameters –M 4, -v 2; [13]). For codominant genotyping, posterior probability is calculated for two possible genotypes (i.e. homozygote or heterozygote) at a given locus using a maximum likelihood approach [14]:
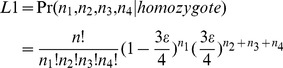
(4)

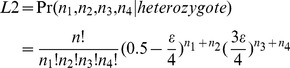
where *n_1_*, *n_2_*, *n_3_* and *n_4_* are the read counts for each of the four possible nucleotides (A, T, C and G), n is the total number of reads and *ε* is the sequencing error rate. The genotype is assigned based on the result of a likelihood ratio test (LRT) between the two most likely hypotheses with one degree freedom. Using a significant level of *α* = 0.05, we assign the most likely genotype at the given locus; otherwise, the genotype is uncalled.

For dominant genotyping, supposing that the cluster depth of the *i* th site for the *j* th progeny is *d_ij_*, this site is genotyped as “presence” if *d_ij_*>*l_d_*, “absence” if *d_ij_* = 0, and “unknown” if *d_ij_*∈(0, *l_d_*), where the threshold *l_d_* is determined using formula (2) with *C* representing the mean sequencing depth of the *i* th site.

### Genotype validation by Sanger sequencing

To verify the genotypes obtained from the first 2b-RAD sequencing dataset, eight codominant and eight dominant markers were randomly selected for Sanger sequencing. The selected marker sequences were mapped to the aforementioned *C. farreri* reference genome to retrieve flanking sequences for primer design. Primers were designed to amplify a fragment (150–300 bp) flanking each target site (primer sequences are provided in Table 3). Each PCR amplification was performed in a 20-µl volume composed of approximately 20 ng genomic DNA, 0.2 µM of each primer, 200 µM of each dNTP, 1.5 mM MgCl_2_, 1 U of Taq DNA polymerase (Takara) and 1× PCR buffer. All cycling programs began with an initial denaturation at 95°C for 5 min, followed by 26–30 cycles of 95°C for 30 s, 60°C for 30 s, 72°C for 30 s and a final extension at 72°C for 5 min. Each PCR product was run on a 1.5% agarose gel to determine the success of the PCR. PCR products amplified from two parents and four progeny were purified using the QIAquick PCR purification kit (Qiagen), and then were sequenced using the Sanger method.

## Supporting Information

Table S1 **Evaluation of the** ***de novo*** **RADtyping approach using a pseudo F_1_ mapping population.** The simulated population was created by crossing two *Arabidopsis* plants with predefined SNPs in their genomes, and was subject to *in silico* sequencing together with their parents at different sequencing depths with sequencing errors enabled. *De novo* codominant and dominant genotyping was evaluated in three key aspects: genotype coverage (a, b), removal of repetitive sites (b, e), and genotyping accuracy (c, f).
(PDF)
