# Supplementary material for: RADtyping: An Integrated Package for Accurate De Novo Codominant and Dominant RAD Genotyping in Mapping Populations
Source: PLoS One. 2013 Nov 21;8(11):e79960. doi: 10.1371/journal.pone.0079960 (PMC3836964; doi:10.1371/journal.pone.0079960)
Supplement: Table S1 — Evaluation of the de novo RADtyping approach using a pseudo F1 mapping population. The simulated population was created by crossing two Arabidopsis plants with predefined SNPs in their genomes, and was subject to in silico sequencing together with their parents at different sequencing depths with sequencing errors enabled. De novo codominant and dominant genotyping was evaluated in three key aspects: genotype coverage (a, b), removal of repetitive sites (b, e), and genotyping accuracy (c, f). (PDF) [file pone.0079960.s001.pdf]

Supporting Information

Table S1. Evaluation of the *de novo* RADtyping approach using a pseudo F_1 mapping population. The simulated population was created by crossing two *Arabidopsis* plants with predefined SNPs in their genomes, and was subject to *in silico* sequencing together with their parents at different sequencing depths with sequencing errors enabled. *De novo* codominant and dominant genotyping was evaluated in three key aspects: genotype coverage (a, b), removal of repetitive sites (b, e), and genotyping accuracy (c, f).

Table S1a. Percentage of ungenotyped loci for codominant genotyping

Progeny \ Parent	10X	20X	30X	40X	50X
5X	0.9739	0.9668	0.9613	0.9622	0.9641
10X	0.3279	0.1435	0.1122	0.1095	0.1103
15X	0.2661	0.0627	0.0487	0.0391	0.0393
20X	0.2548	0.0538	0.0310	0.0240	0.0281
25X	0.2627	0.0510	0.0268	0.0249	0.0193
30X	0.2757	0.0493	0.0276	0.0230	0.0227

Table S1b. Percentage of repetitive loci for codominant genotyping

Progeny \ Parent	10X	20X	30X	40X	50X
5X	0.0407	0.0321	0.0255	0.0169	0.0159
10X	0.0139	0.0136	0.0120	0.0146	0.0165
15X	0.0133	0.0181	0.0201	0.0186	0.0175
20X	0.0174	0.0180	0.0206	0.0179	0.0219
25X	0.0184	0.0213	0.0173	0.0201	0.0217
30X	0.0176	0.0181	0.0188	0.0204	0.0204

Table S1c. Error rate for codominant genotyping

Progeny \ Parent	10X	20X	30X	40X	50X
5X	0.3028	0.2985	0.2758	0.2809	0.2763
10X	0.1243	0.1251	0.1233	0.1232	0.1267
15X	0.0430	0.0495	0.0511	0.0489	0.0497
20X	0.0252	0.0269	0.0283	0.0266	0.0304
25X	0.0189	0.0211	0.0184	0.0214	0.0220
30X	0.0142	0.0162	0.0175	0.0185	0.0186

Table S1d. Percentage of ungenotyped loci for dominant genotyping

Parent / Progeny	10X	20X	30X	40X	50X
5X	0.9901	0.9868	0.9858	0.9865	0.9863
10X	0.2082	0.0940	0.0964	0.0906	0.1014
15X	0.1561	0.0393	0.0359	0.0367	0.0359
20X	0.1543	0.0402	0.0367	0.0364	0.0363
25X	0.1570	0.0400	0.0363	0.0368	0.0370
30X	0.1528	0.0401	0.0371	0.0380	0.0368

Table S1e. Percentage of repetitive loci for dominant genotyping

Parent / Progeny	10X	20X	30X	40X	50X
5X	0.0839	0.0684	0.0588	0.0825	0.0711
10X	0.0055	0.0044	0.0042	0.0048	0.0040
15X	0.0058	0.0043	0.0042	0.0045	0.0045
20X	0.0058	0.0045	0.0044	0.0039	0.0042
25X	0.0051	0.0039	0.0043	0.0041	0.0038
30X	0.0053	0.0041	0.0041	0.0039	0.0039

Table S1f. Error rate for dominant genotyping

Parent / Progeny	10X	20X	30X	40X	50X
5X	0.0546	0.0414	0.0404	0.0486	0.0409
10X	0.0180	0.0180	0.0180	0.0180	0.0173
15X	0.0212	0.0219	0.0213	0.0215	0.0212
20X	0.0215	0.0208	0.0216	0.0206	0.0210
25X	0.0207	0.0208	0.0206	0.0205	0.0199
30X	0.0233	0.0230	0.0237	0.0236	0.0225
